# Decreased expression of CHIP leads to increased angiogenesis via VEGF-VEGFR2 pathway and poor prognosis in human renal cell carcinoma

**DOI:** 10.1038/srep09774

**Published:** 2015-05-29

**Authors:** Chao Sun, Hai-long Li, Hai-rong Chen, Mei-lin Shi, Qing-hua Liu, Zhen-qiang Pan, Jin Bai, Jun-nian Zheng

**Affiliations:** 1 The First Clinical Medical College, Nanjing Medical University, Nanjing, 210029, China; 2 Jiangsu Key Laboratory of Biological Cancer Therapy, Xuzhou Medical College, Xuzhou, 221002, China; 3 Department of Urology, The First Affiliated Hospital of Bengbu Medical College, Bengbu, 233000, China; 4 Department of Occupational Medicine and Environmental Health, School of Public Health, Nanjing Medical University, Nanjing, 210029, China; 5 School of Pathology, Xuzhou Medical College, Xuzhou, 221002, China; 6 Department of Oncological Sciences, Icahn School of Medicine at Mount Sinai, New York, 10029, NY, USA; 7 Jiangsu Center for the Collaboration and Innovation of Cancer Biotherapy, Cancer Institute, Xuzhou Medical College, Xuzhou, 221002, China

## Abstract

CHIP (c-terminal Hsp70-interacting protein) is an E3 ligase which may play different roles in different cancers. The elucidation of the VHL-HIF-1α(hypoxia inducible factor-1α)-VEGF (vascular endothelial growth factor) pathway has led to the development of targeted therapy in renal cell carcinoma (RCC). However, little is known about the role of CHIP and the relationship between CHIP and VEGF-VEGFR2 (VEGF receptor 2) pathway in RCC. In this study, we found that the expression of CHIP was downregulated and significantly correlated with pT status (*P* = 0.022) and TNM stage (*P* = 0.022) in 304 RCC and 35 normal renal tissues using tissue microarray. Moreover, low expression of CHIP is a strong and independent negative prognostic value for RCC. *In vitro*, CHIP negatively regulated RCC cell migration, invasion and angiogenesis. In addition, ELISA tests showed that restoration of CHIP inhibited, while knockdown promoted, the secreted level of VEGF. Furthermore, western blot indicated that the VEGFR2 protein level was reduced after CHIP overexpression. Our findings demonstrate for the first time that CHIP may be involved in RCC angiogenesis through regulating VEGF secretion and expression of VEGFR2. CHIP may serve as promising prognostic biomarker of angiogenesis and may constitute a potential therapeutic target in RCC.

RCC accounts for approximately 3% of all malignancies, and the incidence of RCC is increasing by a rate of approximately 2.5% each year^1^. Metastatic lesions are present in 20–30% of patients at the time of RCC diagnosis, and in 20–30% of patients with preoperative absence of metastasis, metastatic lesions appear after nephrectomy or local recurrence of disease take place^2^. As a result, identification and validation of appropriate biomarkers for prediction or evaluation of prognosis remain important issues.

To grow beyond 1–2 mm in diameter, the tumor requires the emergence and further development of its own blood vessels, which is known as “neoangiogenesis”. RCC is a highly vascular tumor characterized by inactivation of the VHL gene. Its gene product, pVHL, binds the hydroxylated form of HIF-1α ultimately leading to its destruction by the proteasome. In the absence of functional pVHL, HIF-1α accumulates, which causes downstream upregulation of a number of pro-angiogenic factors, including the VEGF^3^. VEGF exerts its biologic effect through interaction with receptors present on the cell surface. These transmembrane tyrosine kinase receptors include VEGFR1, VEGFR2 and VEGFR3. Upon binding of VEGF to the extracellular domain of the receptor, dimerization and autophosphorylation of the intracellular receptor tyrosine kinases occurs and a cascade of downstream proteins are activated. The VEGFR2 are high-affinity VEGF receptors, mediating the majority of VEGF downstream angiogenic effects^4^. It has been shown that blockade of VEGFR2 resulted in inhibition of tumor growth and in abrogation of tumor cell invasion^5^. Importantly, VEGF receptors have been identified on the surface of renal cancer cells, suggesting that VEGF may augment RCC tumor growth through an autocrine loop^6^.

CHIP acts as E3 ligase in combination with cytoplasmic chaperones heat shock protein 70 (HSP70) and HSP90 while mediating the ubiquitination and degradation of various chaperone-bound proteins^7^. CHIP has been demonstrated to regulate a number of oncogenic proteins including receptor tyrosine kinase ErbB2, HIF-1α, estrogen receptor-α (ER-α), and androgen receptor (AR)^8^,^9^,^10^,^11^. Previous studies have indicated that CHIP may play different roles in different human cancers. The phenotypes determined by CHIP should be dependent on the function of its specific targets in a specific type of cancer^12^. However, to date, the function of CHIP in RCC has not yet been reported. In addition, the relationship between CHIP and VEGFR2 in RCC is still unclear.

In this study, we tested the hypothesis that CHIP regulates cell migration, invasion and angiogenesis by inhibiting VEGF signaling pathway in RCC. First, we used a tissue microarray (TMA) of human RCC patients and immunohistochemistry (IHC) to evaluate the expression of CHIP in relation to clinicopathologic features. We further investigated the role of CHIP in RCC cell migration, invasion and angiogenesis. Finally, we studied the relation between CHIP expression and the secreted level of VEGF and VEGFR2 protein expression.

## Results

### CHIP expression is decreased in human RCC

**Figure 1:**
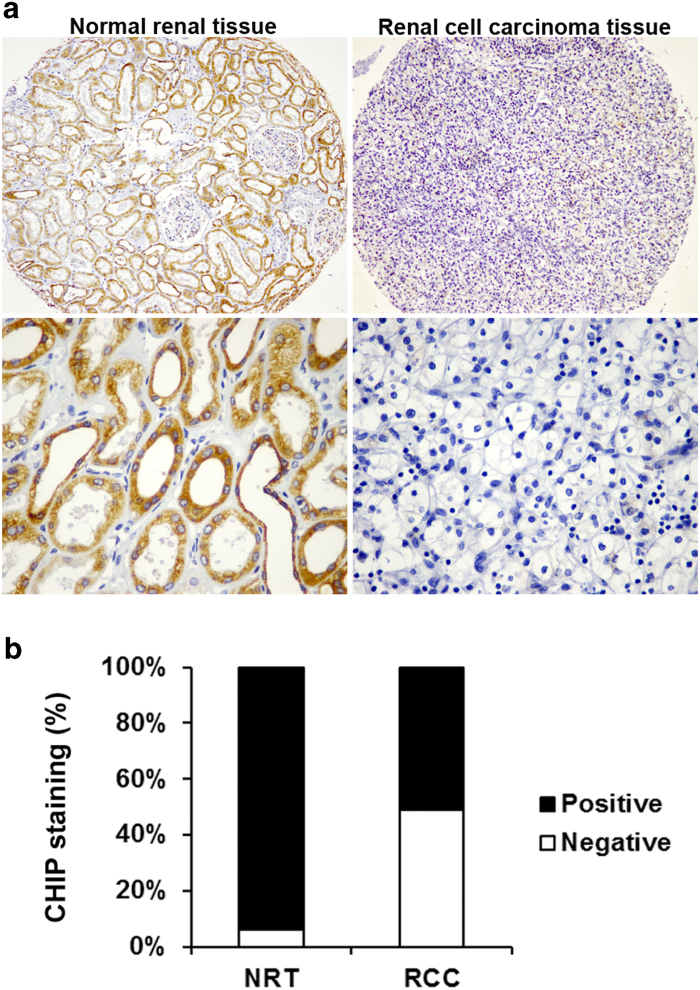
CHIP protein expression in NRT and RCC tissues. (**A**) Representative photographs showed CHIP IHC staining in TMA, which were taken at different magnifications (Top panel ×100, bottom panel ×400). (**B**) CHIP staining was decreased in RCC tissues compared with NRT. Positive CHIP staining was recorded in 94.1% (32/34) and 51.0% (155/304) of the biopsies in NRT and RCC tissue, respectively. (*P* = 0.0001, χ^2^ test).

To investigate whether CHIP expression is changed in RCC, IHC staining was performed in our established TMA slides (Fig. 1A). Positive CHIP staining was recorded in 94.1% (32/34) and 51.0% (155/304) of the biopsies in normal renal tissues (NRT) and RCC tissue, respectively (Fig. 1B).We found that CHIP staining was mainly localized in the cytoplasm and the expression of CHIP was significant lower in the carcinoma tissues than the NRT (*P* = 0.0001).

### Expressions of CHIP correlated with clinicopathological characteristics in RCC patients

The clinicopathologic features of the 304 RCC biopsies were summarized in Table 1. Because TNM stage is an important prognostic marker for RCC patients, we studied if CHIP expression correlates with TNM stage. Two sided Fisher’s exact analysis revealed a significant difference in CHIP expression between TNM stages I and II to IV (*P* = 0.022). CHIP staining was dramatically decreased in TNM stages II to IV compared with stages I. Because TNM stage concludes T-depth of invasion, N-lymph node metastasis, and M-distant metastasis, we analyzed the correlation between CHIP expression and these clinicopathologic parameters. The analysis indicated a significant correlation between CHIP expression and depth of invasion when comparing pT_1_ versus pT_2_-pT_4_ (*P* = 0.022). We did not find significant correlation between CHIP expression with other clinicopathologic variables, including age, gender and tumor size.

### Decreased CHIP expression correlates with poor patient survival

**Figure 2:**
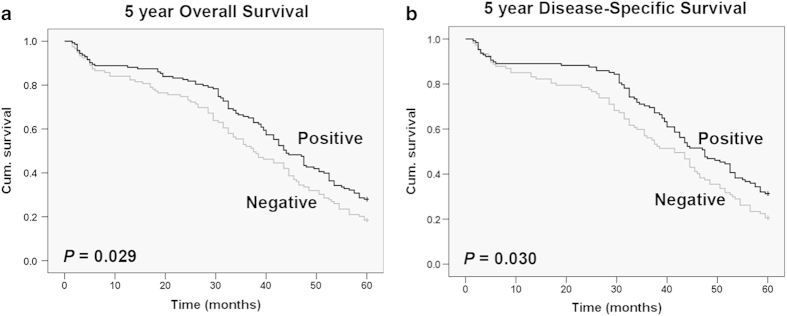
CHIP expression associated with both 5-year OS and DSS in RCC patients. (**A**) Kaplan-Meier curves showed that the 5-year OS dropped from 28.0% in patients with positive CHIP expression to 18.5% in those with negative expression (*P* = 0.029, log-rank test). (**B**) Kaplan-Meier curves showed that the 5-year DSS dropped from 31.2% in patients with positive CHIP expression to 20.6% in those with negative expression (*P* = 0.030, log-rank test). Cum., cumulative.

To study whether reduced CHIP staining in RCC patients correlates with a worse prognosis, Kaplan-Meier survival curves were plotted using 5-year overall survival (OS) and disease-specific survival (DSS) (n = 304, follow-up time, 60 months) to compare the patients with positive CHIP staining to those with negative staining (Fig. 2). Our data revealed that positive CHIP staining correlated with both 5-year OS and DSS in RCC (*P* = 0.029 and *P* = 0.030, respectively, log-rank test). The 5-year OS dropped from 28.0% in patients with positive CHIP expression to 18.5% in those with negative expression, and the 5-year DSS dropped from 31.2% in patients with positive CHIP expression to 20.6% in those with negative expression.

Furthermore, we examined whether CHIP expression is an independent prognostic marker for RCC. We performed univariate and multivariate Cox regression analysis including CHIP expression, age, tumor size and TNM stage to study the effects of CHIP on patient survival in RCC. The univariate Cox regression analysis showed that CHIP expression was an independent prognostic marker for RCC patients (hazard ratio, 0.738; 95% confidence interval, 0.559–0.974; *P* = 0.032; Supplementary Table S1). In multivariate Cox regression analysis, we found that similar to TNM stage, which has been widely accepted as an important prognostic factor for RCC patients, CHIP expression was also an independent prognostic factor for 5-year survival (hazard ratio, 0.739; 95% confidence interval, 0.595–1.074; *P* = 0.021; Supplementary Table S2). Because 5-year patient survival is widely used to predict outcome in RCC, our results clearly indicated that low CHIP expression is associated with poor prognosis, suggesting that CHIP may serve as a molecular prognostic marker for this aggressive disease.

### CHIP regulates RCC cells migration and invasion *in vitro*

**Figure 3:**
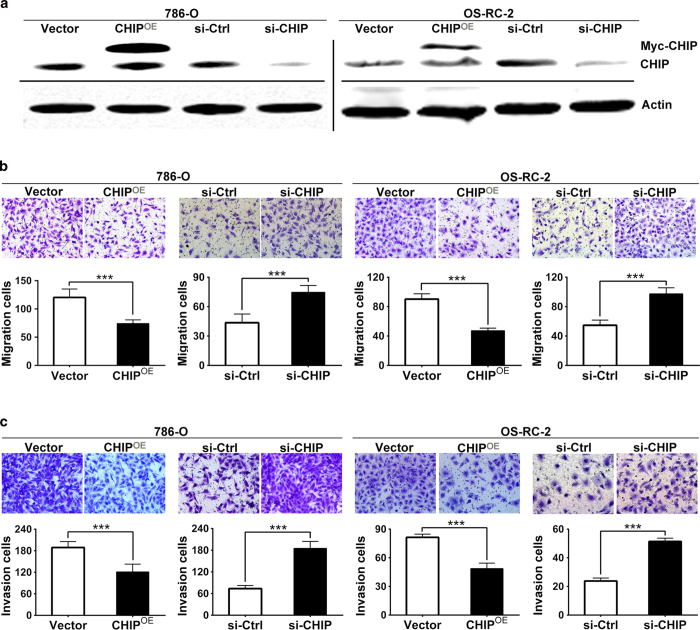
CHIP negatively regulated cell migration and invasion in 786-O and OS-RC-2 cell lines. (**A**) WB showed that twenty-four hours or forty-eight after transfection, CHIP protein was significantly overexpressed or knockdown in cancer cells, respectively. Actin was used as an internal control. (**B**) Cell migration assay was performed after reintroduction or knockdown of CHIP in 786-O and OS-RC-2 cells. The ability of cell migration was decreased after CHIP overexpression. In contrast, CHIP knockdown promoted cell migration. (**C**) Matrigel cell invasion assay was performed after reintroduction or knockdown of CHIP in 786-O and OS-RC-2 cells. The ability of cell invasion was decreased after CHIP overexpression while CHIP knockdown promoted cell invasion. The data are presented as mean ± s.e.m. for triplicate determinations. *** *P* < 0.001.

Because low CHIP expression is associated with poor prognosis, supporting CHIP may play important roles in one or more steps of tumor metastasis, we investigated the involvement of CHIP in RCC cells migration and invasion. To determine the effect of CHIP overexpression or knockdown on RCC cells migration and invasion, we transiently transfected 786-O and OS-RC-2 cells with Myc-control and Myc-CHIP plasmids or control siRNA and CHIP siRNA, respectively. Twenty-four hours or forty-eight after transfection, CHIP protein was considerably overexpressed or knockdown in cancer cells, respectively (Fig. 3A). In cell migration assay, we found that the ability of cell migration was drastically decreased after CHIP overexpression in both 786-O and OS-RC-2 RCC cell line. In contrast, knockdown CHIP promoted cell migration (Fig. 3B). Meanwhile, the results of the cell invasion assay corresponded with the cell migration assay (Fig. 3C).

### Expression of CHIP inhibits angiogenesis *in vitro*

**Figure 4:**
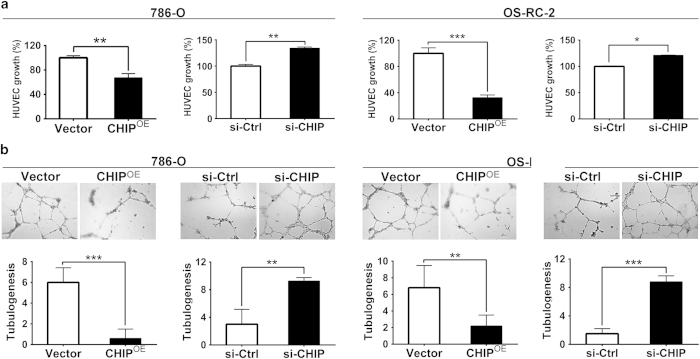
CHIP negatively regulated angiogenesis in 786-O and OS-RC-2 cell lines. (**A**) HUVEC growth assay was performed to detect the HUVECs proliferation. CHIP overexpression significantly inhibited proliferation of HUVECs. Nevertheless, CHIP knockdown significantly promoted proliferation of HUVECs. (**B**) Representative pictures were taken *in situ* for tube formation in the supernatant of 786-O and OS-RC-2 cells (×100). The degree of tube formation was assessed as the number of tube. The average number of complete tubular structures formed by HUVECs from CHIP overexpression in the 786-O and OS-RC-2 cells led to 90% and 68% decrease, respectively; whereas increased 3.08- and 5.87-fold with knockdown of CHIP, respectively. All experiments were carried out in triplicate. Data are shown as mean ± s.e.m. ***P < 0.001, **P < 0.01, *P < 0.05.

Mutations of VHL gene are present in 60% of RCC patients. The VHL gene inhibits hypoxia inducible genes including proteins involved in angiogenesis, which plays important roles in the progression of RCC^2^. To test the effect of CHIP expression in RCC angiogenesis, we overexpressed or knocked down CHIP in 786-O and OS-RC-2 cells, respectively. The condition medium containing secreted cytokines was collected from these either overexpressing or knockdown RCC cells and used for HUVECs growth and tube formation assays. In the HUVECs growth assay, we found that conditioned medium from 786-O and OS-RC-2 cells overexpressing CHIP significantly inhibited proliferation of endothelial cells (*P* = 0.017 and *P* = 0.019, respectively). Nevertheless, conditioned medium with CHIP knockdown substantially promoted proliferation of endothelial cells (Fig. 4A). In addition, as shown in Fig. 4B, the average number of complete tubular structures formed by HUVECs in conditioned medium from CHIP overexpression led to 90% and 68% decrease, respectively; whereas increased 3.08- and 5.87-fold with knockdown of CHIP, respectively.

### CHIP suppresses secreted level of VEGF and protein expression of VEGFR2 in RCC cells

**Figure 5:**
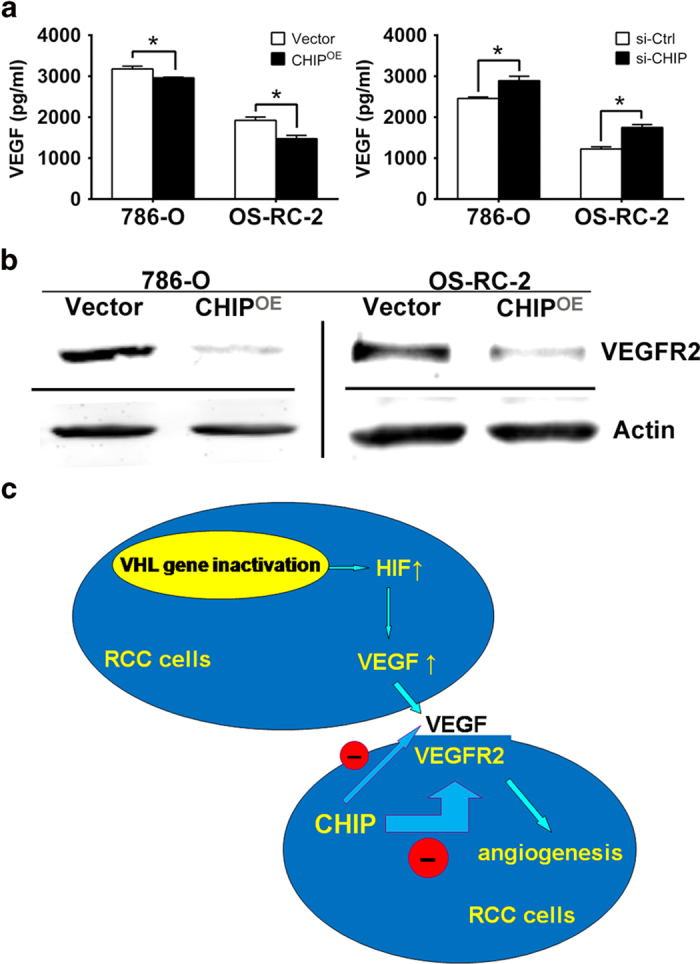
CHIP suppresses the secreted level of VEGF and protein expression of VEGFR2 in RCC cells. (**A**) The secreted level of VEGF was determined by ELISA assay. VEGF secretion level was negatively regulated by CHIP in both 786-O and OS-RC-2 cells, respectively. All experiments were carried out in triplicate. (**B**) Representative WB analysis of the relative protein levels of VEGFR2 in CHIP restoration and control group for both 786-O and OS-RC-2 cell lines. VEGFR2 protein level was dramatically reduced after CHIP restoration. (**C**) Model of mechanism of CHIP inhibiting RCC angiogenesis. CHIP decreased the secreted level of VEGF, meanwhile increased the degradation of VEGFR2 and downregulated the protein level of VEGFR2 on cell membrane. Data are shown as mean ± s.e.m. *P < 0.05.

RCC is characterized by the VHL loss or its inactivation, and the process of angiogenesis is controlled by the VHL-HIF(1α)-VEGF-VEGFR2 signal pathway^13^. Here we investigated whether the role of CHIP on angiogenesis was mediated by regulating VEGF secretion and VEGFR2 expression. Using ELISA test, we first observed if VEGF secretion level was regulated by CHIP in RCC cells. As shown in Fig. 5A, VEGF secretion level was negatively regulated by CHIP in both 786-O and OS-RC-2 cells, respectively. Moreover, we performed western blot (WB) to measure the VEGFR2 expression in RCC cells. Our data showed that the VEGFR2 protein level was dramatically reduced in 786-O and OS-RC-2 cells after being transfected with Myc-CHIP (Fig. 5B).

## Discussion

As a member of the E3 ubiquitin ligases, CHIP associates with molecular chaperones (Hsp70 or Hsp90), ubiquitinating the client proteins, and is demonstrated to be involved in tumorigenesis in several malignancies^12^. For example, CHIP regulates oncogenic proteins such as steroid receptor coactivator 3 in breast cancer^14^, and AR in prostate cancer^8^. However, there has been no attempt to evaluate the expression and function of CHIP in human RCC to date.

In the present study, we used a RCC TMA containing 304 tumor biopsies and 35 NRT with robust clinical data to investigate the role of CHIP in RCC development. Our data demonstrated that CHIP expression of RCC was significantly downregulated compared with the NRT and reduced CHIP expression significantly correlated with TNM stage, depth of invasion and poorer survival of RCC patients. Furthermore, univariate and multivariate Cox proportional hazards regression analysis showed that low CHIP expression was a strong independent negative prognostic factor after adjusted by age, tumor size, pT status and TNM stages. Such information indicates that CHIP may play a significant role in the progression of RCC and testing for the presence of CHIP may help identify patient subgroups at high risk for poor disease outcome.

Reduced expression of some tumor suppressive genes may play a role in cell migration and invasion^15^,^16^. Combined with the TMA results, we supposed that CHIP may inhibit RCC cell migration and invasion abilities. Surprisingly, we found that downregulation of CHIP accelerates RCC cell migration as well as invasion through matrigel-coated chamber *in vitro*, whereas the cell invasion and migration abilities are dramatically suppressed when CHIP was overexpressed, respectively. As increased cell migration is a crucial step in cancer metastasis, these findings implied that CHIP may regulate the metastatic potential of RCC. Previous studies have shown that increased expression of matrix metalloproteinase-2 (MMP-2) and MMP-9 is associated with enhanced tumor invasion and metastasis^17^. We examined the levels of MMP-2 and MMP-9 after CHIP overexpression or knockdown by WB and gelatin zymography experiments. Unexpectedly, the results showed that the changes in CHIP levels did not affect the expression of MMP-2 and MMP-9 (see Supplementary Fig. S3 and Supplementary Methods S4). This implied that CHIP may regulate the invasive ability of RCC cells through other mechanisms, and further studies are needed to address the question.

The essential role of the formation of a new vasculature for tumor progression has been fully recognized in the last decade^18^,^19^. Meanwhile, RCC is a highly vascular tumor which originates from the proximal tubule cells of nephrons. Angiogenesis is a multi-step process, which includes endothelial cell proliferation, migration and the formation of blood vessels^20^. Here, we provided a novel function of CHIP in regulating angiogenesis in RCC cells. Using HUVEC growth assay and HUVEC tube formation assay, we found that the transfection of Myc-CHIP reduced, while CHIP knockdown increased, the capacity of proliferation and tube formation of HUVECs, suggesting that CHIP significantly affected angiogenic potential of RCC cells *in vitro*.

The majority of RCCs have clear cell histology, which is characterized by genetic and epigenetic inactivation of the VHL gene. Loss of VHL function upregulates HIF-1α, a transcription factor that leads to an overexpression of VEGF and drives tumor angiogenesis^21^. Otherwise, our results showed that CHIP did not affect the protein level of HIF-1α(see Supplementary Fig. S5). VEGF has been characterized with regard to multiple effects relevant to the generation and preservation of tumor vasculature. These effects include induction of endothelial cell division and migration, promotion of endothelial cell survival through protection from apoptosis, and reversal of endothelial cell senescence^22^,^23^. Of the numerous angiogenic factors discovered thus far, VEGF has been identified as a key mediator of tumor angiogenesis^24^. VEGFR2 is the major mediator of the mitogenic and permeability enhancing effects of VEGF^4^. Previous studies found the increased HIF(1α)-VEGF expression was correlated to adverse survival of RCC^25^,^26^. This led to the development of VEGF signaling pathway inhibitors which have been shown to benefit patients in randomized phase III clinical trials. Currently, there are five angiogenesis inhibitors approved by the United States Food and Drug Administration for treatment of advanced and metastatic RCC, four VEGFR2 targeted tyrosine kinase inhibitors (TKIs), including sorafenib, sunitinib, axitinib and pazopanib, and one anti-VEGF monoclonal antibody, bevacizumab^27^,^28^,^29^,^30^,^31^. Therefore, we hypothesized that CHIP may suppress RCC angiogenesis through inhibiting VEGF-VEGFR2 pathway. We detected the secreted level of VEGF after CHIP transfection using ELISA technology. Our data showed that VEGF secretion was increased by knockdown of CHIP, while decreased by overexpression of CHIP, respectively. These data indicated that CHIP regulates endothelial cell proliferation and blood vessel formation through controlling the VEGF secretion. Furthermore, the results of WB have shown that the expression of VEGFR2 was dramatically decreased after overexpression of CHIP, thus we speculated that CHIP may be involved in the degradation of VEGFR2.

In summary, decreased expression of CHIP in RCC speciments constitute a strong unfavorable prognostic factor for RCC patients. Downregulation of in RCC cells inhibits migration, invasion, proliferation and tube formation of cocultured endothelial cells by decreasing VEGFR2 expression and VEGF secretion (Fig. 5C). Our observations support the hypothesis that CHIP interacts with VEGF-VEGFR2 signaling pathway. We also show that decreased expression of CHIP in RCC cells mat trigger angiogenesis as well as cell migration and may be used as a promising prognostic marker for RCC patients. Although the development of TKIs targeting VEGFR2 opens a new door for advanced RCC patients, the huge costs and side effects, such as neutropenia, anemia, increased creatinine, thrombocytopenia and hand-foot syndrome may become the stumbling block of these treatments^32^. The search for a new and better therapy via new targets for metastatic RCC is still needed. Our results provided the first *in vitro* evidences that targeting CHIP might represent a new therapy to suppress RCC progression. We hope these findings might have shed light on future directions for identification of novel biomarkers for RCC and the development of targeted drugs.

## Methods

### Ethics Statement

This study was performed under a protocol approved by the Institutional Review Boards of Affiliated Hospital of Xuzhou Medical College and all examinations were performed after obtaining written informed consents. All experiments involving human subjects were performed in accordance with relevant guidelines and regulations.

### Patients and samples

The study material consists of 304 consecutive cases of RCC and 35 cases of NRT, from Affiliated Hospital of Xuzhou Medical College, between 2005 and 2008. All these patients were treated with surgery only or with postoperative adjuvant therapy. The patients’ clinicopathologic information was obtained from the archive of the pathology department and confirmed by the medical record of the hospital. Five-year clinical follow-up results were available for 262 patients.

### IHC of TMA

IHC was performed as described before^33^. According to the streptavidin-peroxidase (Sp) method using a standard Sp Kit (Zhongshan biotech, Beijing, China). The TMA slides were incubated with rabbit anti-CHIP antibody (1:100) (Bethyl Laboratories, Montgomery, USA) overnight at 4 °C, and diaminobenzidine (DAB; Zhongshan Biotech, Beijing, China) was used to produce a brown precipitate. The immunoreactivity was assessed blindly by two independent observers using light microscopy (Olympus BX-51 light microscope), and the image was collected by Camedia Master C-3040 digital camera. The expression of CHIP was graded as positive when 5% of tumor cells showed immunopositivity. Biopsies with < 5% tumor cells showing immunostaining were considered negative.

### Cell lines and transfection

Human RCC cell lines 786-O and OS-RC-2 were purchased from the Shanghai Institute of Biochemistry and Cell Biology, Chinese Academy of Sciences (Shanghai, China). Human umbilical vascular endothelial cells (HUVECs) were obtained from Key-GEN biotech (Nanjing, China). 786-O, OS-RC-2 and HUVEC cells were cultured in RPMI1640 medium supplemented with 10% fetal calf serum (Invitrogen, Shanghai, China). Cells were in a 37 °C humidified incubator with 5% CO_2_. The CHIP overexpression plasmids were obtained from Dr. Shouyu Wang (Nanjing Medical University, Jiangsu, China). Non-specific control siRNA or CHIP siRNA was purchased from GenePharma biotech (Shanghai, China). Transfection of the plasmids into the renal carcinoma cells were carried out using Lipofectamine 2000 transfection reagent (Invitrogen, Shanghai, China) following the manufacturer’s protocol. Transfection of the non-specific control siRNA or CHIP siRNA were carried out using siLentFect Lipid Reagent (Bio-Rad, Hercules, CA, USA) according to the manufacturer’s instructions.

### Migration assay

Cell migration was determined by using a modified two chamber migration assay with a pore size of 8 μm^34^. For migration assay, 3 × 10^4^ 786-O and OS-RC-2 cells were seeded in serum-free medium in the upper chamber. After 12 h incubation at 37 °C, cells in the upper chamber were carefully removed with a cotton swab and the cells that had traversed the membrane were fixed in methanol and stained with leucocrystal violet. The number of migration cells was determined by counting the leucocrystal violet stained cells. For quantification, cells were counted under a microscope in five fields (up, down, median, left, right. × 200).

### Invasion assay

The invasion assay was performed using a modified two chamber plates with a pore size of 8 μm^15^. The transwell filter inserts were coated with matrigel (BD Biosciences, NJ, USA). 5 × 10^4^ 786-O and OS-RC-2 cells were seeded in serum free medium in the upper chamber. After 24 h incubation at 37 °C, no invasive cells were gently removed from the top of the matrigel with a cotton-tipped swab. Invasive cells at the bottom of the matrigel were fixed in methanol, stained with leucocrystal violet and counted.

### HUVECs growth assay

The HUVECs growth was assayed using cell counting kit-8 (CCK-8) purchased from Beyotime Institute of Biotechnology (Nanjing, China)^16^. In brief, 6×10^3^ HUVECs suspended in 100 μl conditioned medium from control, CHIP overexpression or CHIP siRNA cells in a 96-well culture plate, respectively. Then HUVECs were incubated at 37 °C in a humidified atmosphere containing 5% CO_2_ for 24 h. Then, cell proliferation was detected according to the manufacturer’s instructions.

### Endothelial cell tube formation assay

Transfected 786-O and OS-RC-2 cells (1 × 10^6^) were cultured in 6-well plate with fresh complete medium for 24 h, and the medium was collected and centrifuged to remove any cell debris before its use as a conditioned medium. 48-well plate was coated with matrigel and kept in 37 °C for 30 min. Then, 2 × 10^4^ HUVECs were suspended in 100 μl conditioned medium and applied to the pre-coated 48-well plate. After incubation at 37 °C for another 24 h, the number of capillary-like tubes from four randomly chosen fields was counted ( × 100).

### ELISA for VEGF

786-O and OS-RC-2 cells were plated in 6-well tissue culture plates at a density of 1 × 10^6^ cells per well. Then, cells were transfected with CHIP overexpression or CHIP siRNA with serum starvation. The supernatants were collected 24 h after transfection. VEGF concentration was determined using Quantikine ELISA kits according to the manufacturer’s instructions (Westang biotech, Shanghai, China).

### WB analysis

For cancer cells, twenty-four hours (overexpression) or forty-eight (RNAi) after transfection, cells were harvested from the plates. Then aliquots of cell extracts were separated on a 10% SDS-polyacrylamide gel. The proteins were then transferred to nitrocellulose membrane and incubated overnight at 4 °C with the following antibodies: rabbit anti-CHIP (1: 1000; Cell Signaling Technology, Beverly, MA, USA), rabbit anti-VEGFR2 (1: 500; Bioworld Technology, St. Louis Park, MN, USA) and mouse anti-β-actin (1: 1000; Boster Biotechnology, Wuhan, China). Membranes were then washed and incubated with secondary antibody (1: 2000; goat anti-rabbit and goat anti-mouse IgG) for 2 h. Membranes were then washed and scanned on the Odyssey Two-Color Infrared Imaging System (LI-COR Biotechnology, Lincoln, Nebraska, USA). Each blot was repeated three times.

### Statistical analysis

For TMA, statistical analysis was performed with SPSS 20.0 software (SPSS, Chicago, IL). Data are expressed as the means ± SEM. Two-factor analysis of variance procedures and the Dunnett’s *t* test were used to assess differences within treatment groups. The association between CHIP staining and the clinicopathologic parameters of the RCC patients, including age, gender, tumor size, pT status and TNM stage, was evaluated by χ^2^ test. The Kaplan-Meier method and log-rank test were used to evaluate the correlation between CHIP expression and patient survival. Univariate or multivariate Cox proportional hazards regression models were performed to estimate the crude hazard ratios (HRs) and their 95% confidential intervals (CIs). For cell migration, invasion, HUVECs tube formation, VEGF ELISA and CCK-8 cell proliferation assays, statistical analysis was performed with GraphPad Prism 6.0 software, Student *t* test was used. Differences were considered significant when *P* < 0.05.

## Additional Information

**How to cite this article**: Sun, C. *et al.* Decreased expression of CHIP leads to increased angiogenesis via VEGF-VEGFR2 pathway and poor prognosis in human renal cell carcinoma. *Sci. Rep.* **5**, 9774; doi: 10.1038/srep09774 (2015).

## Supplementary Material

Supplementary Information

## Figures and Tables

**Table 1:** CHIP staining and clinicopathological characteristics of 304 renal cancer patients.

	**CHIP staining**			
**Variables**	**Negative (%)**	**Positive (%)**	**Total**	P*
All cases	154 (34.7)	150 (49.3)	304	
				
Age				
≤ 56 years	69 (47.6)	76 (52.4)	145	0.182
> 56 years	85 (53.5)	74 (46.5)	159	
				
Gender				
Male	100 (49.5)	102 (50.5)	202	0.328
Female	54 (52.9)	48 (47.1)	102	
				
Tumor size				
≤ 7 cm	114 (48.3)	122 (51.7)	236	0.082
> 7 cm	40 (58.8)	28 (41.2)	68	
				
pT status				
pT_1_	92 (46.2)	107 (53.8)	199	0.022
pT_2-_ pT_4_	62 (59.0)	43 (41.0)	105	
				
pN status				
pN_0_	142 (51.1)	136 (48.9)	278	0.123
pN_1-_ pN_3_	7 (35.0)	13 (65.0)	20	
				
pM status				
pM_0_	132 (51.8)	123 (48.2)	255	0.332
pM_1_	3 (37.5)	5 (62.5)	8	
				
TNM stage				
I	82 (45.6)	98 (54.4)	180	0.022
II-IV	54 (59.3)	37 (40.7)	91	

^*^Two sided Fisher's exact tests.
