# Supplementary material for: Decreased expression of CHIP leads to increased angiogenesis via VEGF-VEGFR2 pathway and poor prognosis in human renal cell carcinoma
Source: Sci Rep. 2015 May 29;5:9774. doi: 10.1038/srep09774 (PMC4448523; doi:10.1038/srep09774)
Supplement: Supplementary Information [file srep09774-s1.pdf]

Decreased expression of CHIP leads to increased angiogenesis via VEGF-VEGFR2 pathway and poor prognosis in human renal cell carcinoma

Chao Sun[1, 2, 4, *], Hai-long Li[1, 2, *], Hai-rong Chen[6, *], Mei-lin Shi[2], Qing-hua Liu[2, 3], Zhen-qiang Pan[2, 5], Jin Bai [2, 5], Jun-nian Zheng [1, 2]

1 The First Clinical Medical College, Nanjing Medical University, Nanjing, 210029, China; **2** Jiangsu Key Laboratory of Biological Cancer Therapy, Xuzhou Medical College, Xuzhou, 221002, China; **3** School of Pathology, Xuzhou Medical College, Xuzhou, 221002, China; **4** Department of Urology, The Third the People's Hospital of Bengbu, Bengbu, 233000, China; **5** Department of Oncological Sciences, Icahn School of Medicine at Mount Sinai, New York, 10029, NY, USA; **6** Department of Occupational Medicine and Environmental Health, School of Public Health, Nanjing Medical University, Nanjing, 210029, China.

Correspondence to: Jun-nian Zheng, E-mail: jnzheng@xzmc.edu.cn

[*]These authors contributed equally to this paper.

Supplementary Table S1: Univariate Cox proportional regression analysis on 5-year overall and disease-specific survival of 304renal cancer patients.

Variable*	Overall survival			Disease-specific survival		
	Hazard ratio	95% CI†	*P**	Hazard ratio	95% CI†	*P**
CHIP						
Negative	1.000		0.032	1.000		0.033
positive	0.738	0.559-0.974		0.722	0.536-0.974	
Age						
≤56 years	1.000		0.486	1.000		0.619
>56years	1.103	0.836-1.455		1.079	0.800-1.453	
Tumor size						
≤7 cm	1.000		0.011	1.000		0.010
>7 cm	1.422	1.014-1.994		2.040	1.793-2.764	
pT status						
pT_1	1.000		0.010	1.000		0.009
pT_{2-} pT_4	1.462	1.095-1.952		2.299	1.943-2.790	
pN status						
pN_0	1.000		0.006	1.000		0.004
pN_{1-} pN_3	2.413	1.544-3.080		2.445	2.020-3.036	
TNM stage						
I	1.000		0.009	1.000		0.008
II-IV	1.497	1.106-2.026		1.933	1.369-2.628	

**P*values are from Log-rank test.

† CI: confidence interval.

Supplementary Table S2: Multivariate Cox regression analysis on 5-year overall and disease-specific survival of 304 renal cancer patients.

Variable*	Overall survival			Disease-specific survival		
	Hazard ratio	95% CI†	*P*	Hazard ratio	95% CI	*P*
CHIP	0.739	0.595 to 1.074	0.021	0.706	0.560 to 0.947	0.024
Age	1.021	0.762 to 1.369	0.890	1.005	0.736 to 1.372	0.975
Tumor size	1.809	1.369 to 2.725	0.012	1.815	0.911 to 2.807	0.014
TNM stage	1.659	0.996 to 2.138	0.019	1.698	1.344 to 1.934	0.023

*Coding of variables: CHIP was coded as 1 (negative), and 2 (positive). Age was coded as 1 (≤56 years), and 2 (>56 years). Tumor size was coded as 1 (≤7 cm), and 2 (>7 cm). TNM stage was coded as 1 (I), and 2 (II - IV).

† CI: confidence interval.

Revised Supplementary Fig. S3: CHIP levels did not affect the expression of MMP-2 and MMP-9. A Western blot analysis of the relative protein levels of MMP-2,MMP-9 and Actin after CHIP overexpression or knockdown for both 786-O and OS-RC-2 cell lines. B Gelatin zymography analysis of the enzyme activity of MMP-2 and MMP-9 after CHIP overexpression or knockdown for both 786-O and OS-RC-2 cell lines.

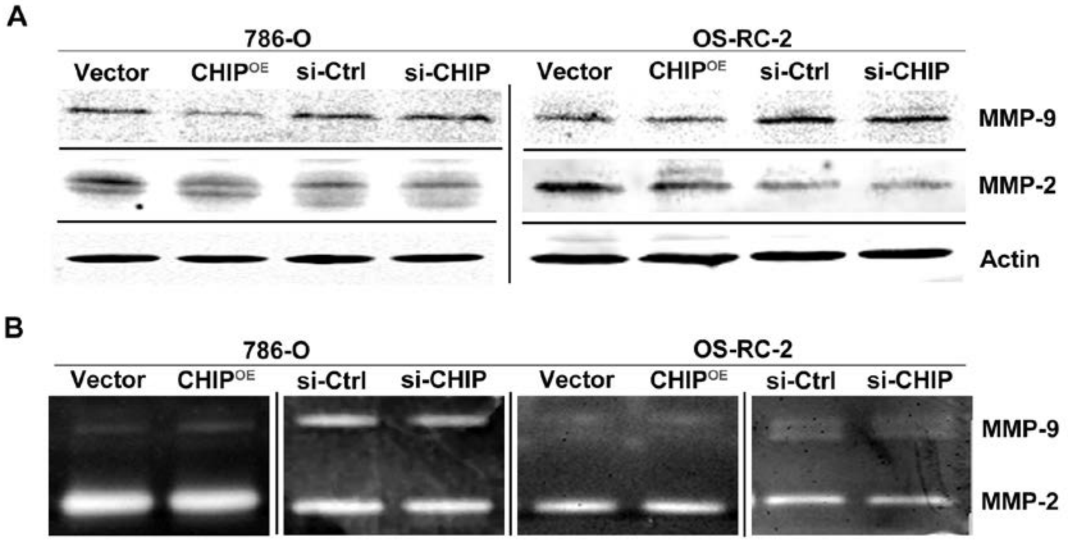

Supplementary Methods S4:

Gelatin zymography: Transfected 786-O and OS-RC-2 cells (1×10^6) were cultured in 6-well plate with fresh complete medium for 24 h, and the mediumwas collected and centrifuged to remove any cell debris before itsuse as a conditioned medium.15μg of the proteins was loaded in non-redenaturingconditions on a 10% polyacrylamide gel containing0.1% gelatin (Sigma, St. Louis, USA). After electrophoresis, gelswere soaked in 2.5% Triton X-100 for 45 minutes with singlechange of detergent solution. Gels were incubated for 36 h at37℃ in substrate buffer (50 mM Tris-HCl, pH 7.5, 5 mM CaCl2,and 0.02% NaN3), stained with 0.05% Coomassie brilliant blue G-250 (Sigma, St. Louis, USA), and destained in 10% acetic acid and20% methanol. Gels were then scanned on the Odyssey Two-Color Infrared Imaging System (LI-COR Biotechnology, Lincoln, Nebraska, USA).

Revised Supplementary Fig. S5: CHIP did not affect the protein expression of HIF-1α.Western blot analysis of the protein levels of HIF-1α and Actin after CHIP overexpression or knockdown for both 786-O and OS-RC-2 cell lines.

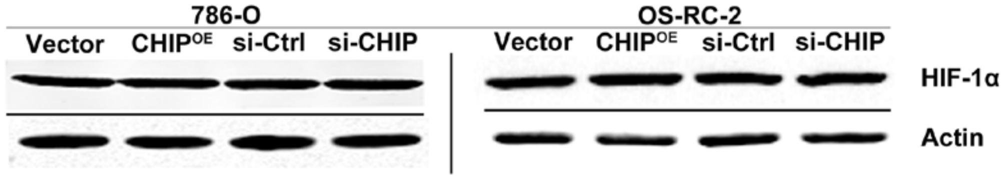

Revised Fig. 3

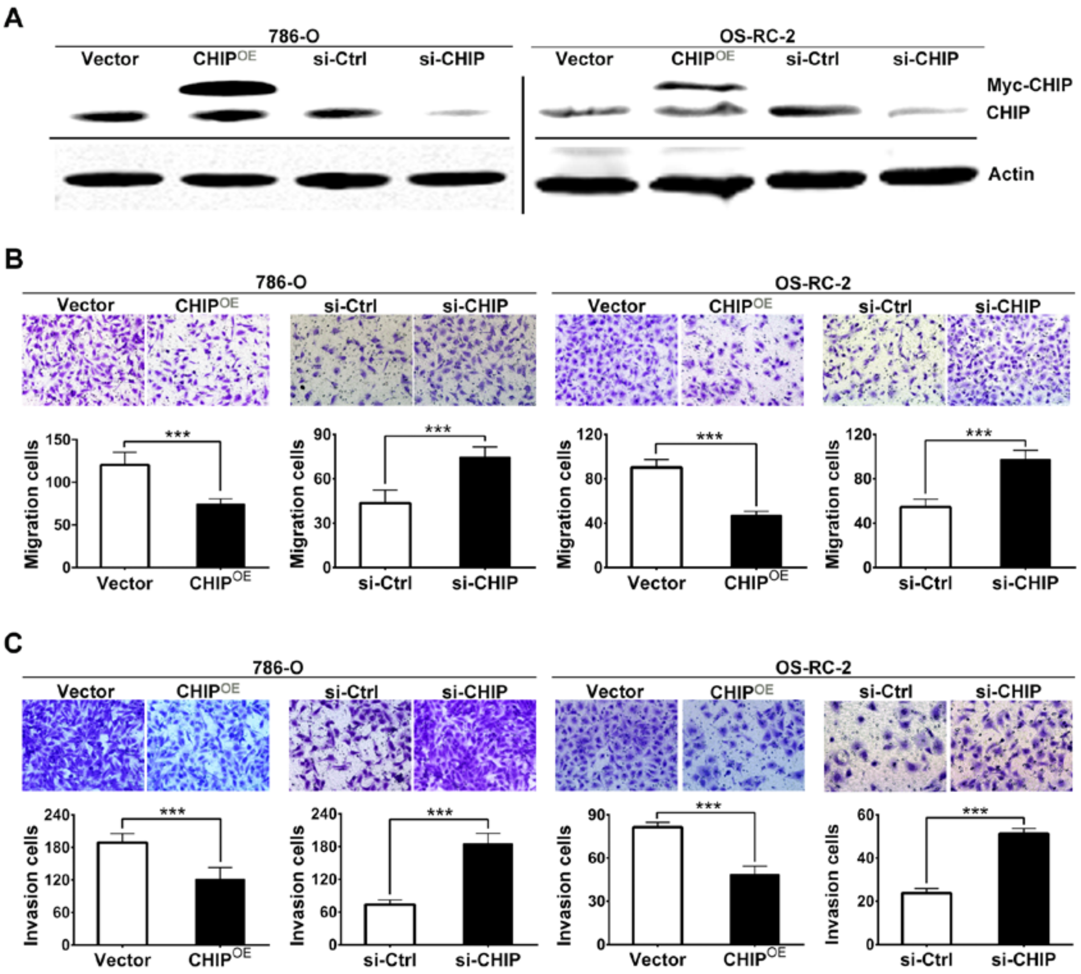

Revised Fig. 5

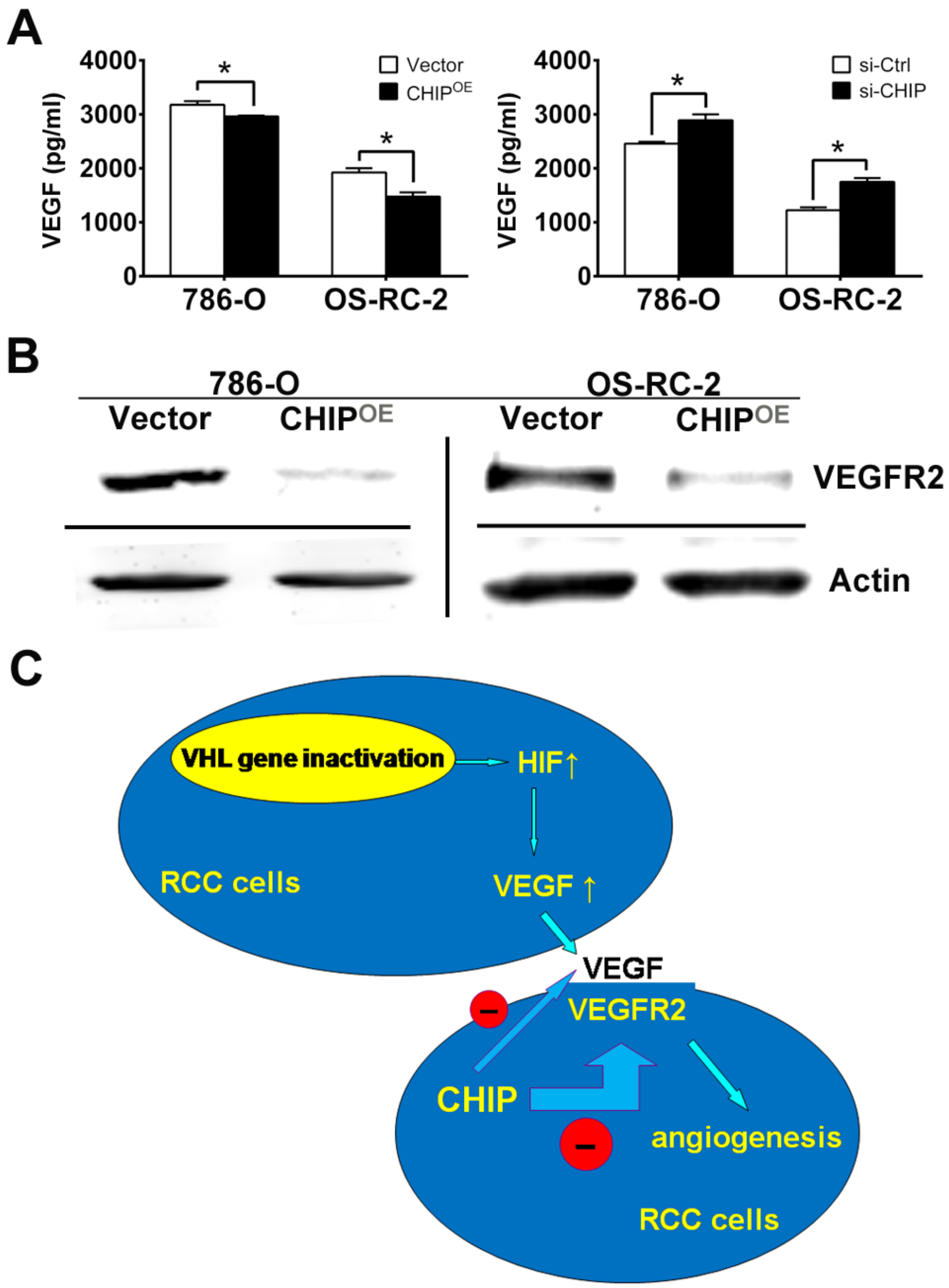

Unprocessed original scans for all of the blots:

All image acquisition tools: Odyssey Two-Color Infrared Imaging System

(LI-COR Biotechnology, Lincoln, Nebraska, USA)

All image processing software packages: Adobe Photoshop CS5.0

Figure 3

786-O

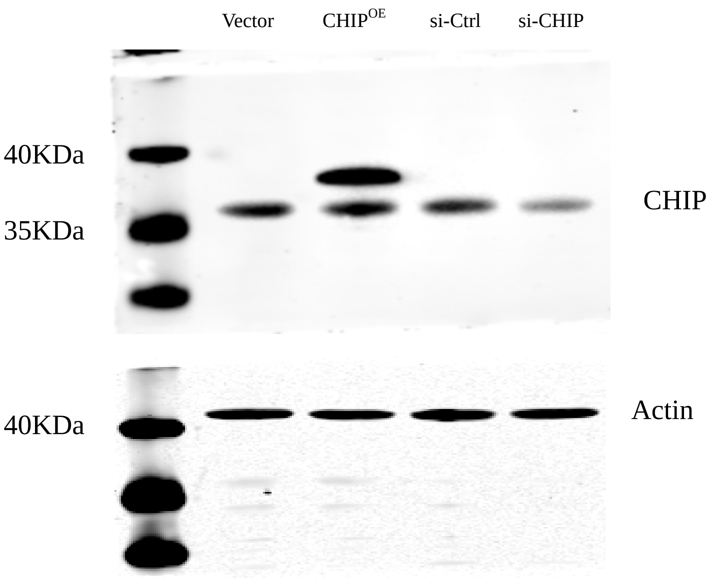

OS-RC-2

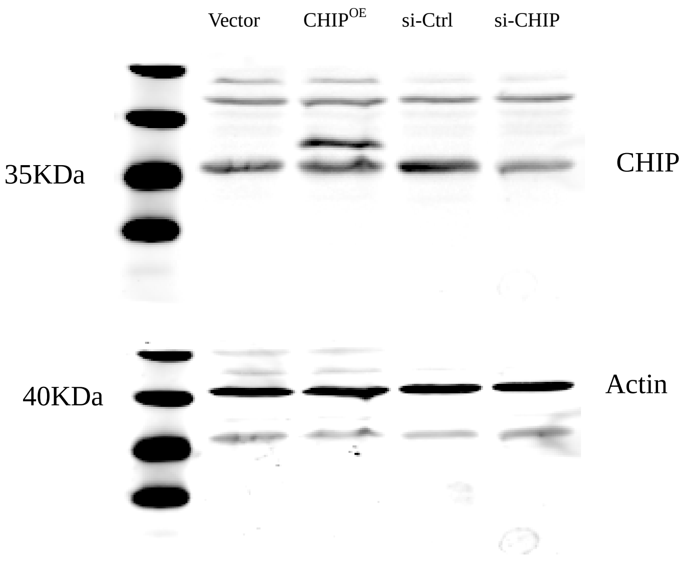

Figure 5

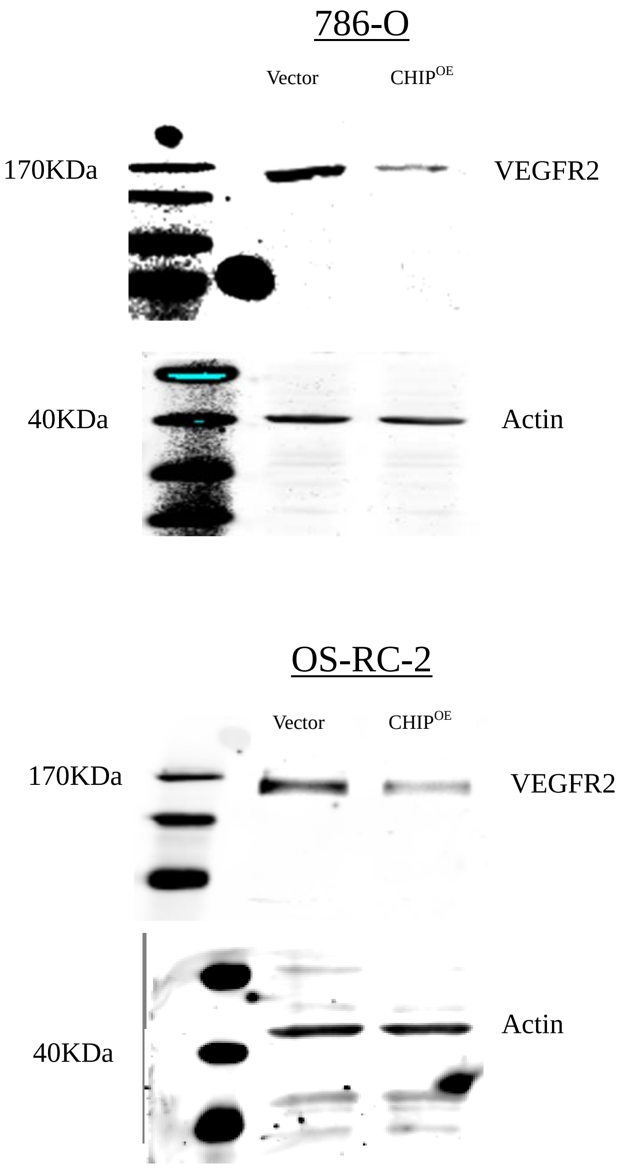
786-O
Vector
CHIP^{OE}
170KDa
VEGFR2
40KDa
Actin
OS-RC-2
Vector
CHIP^{OE}
170KDa
VEGFR2
40KDa
Actin

Figure S3

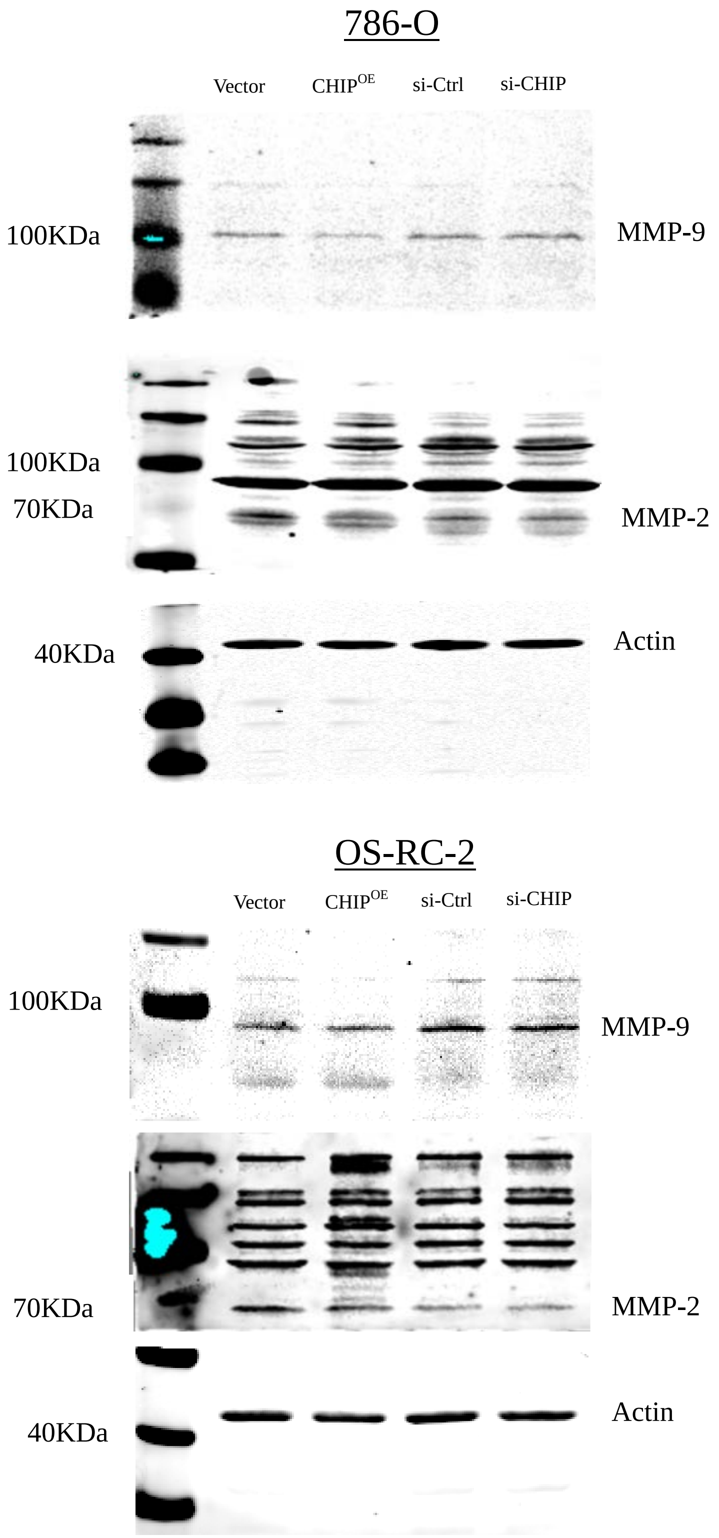
786-O
Vector
CHIPOE
si-Ctrl
si-CHIP
100KDa
MMP-9
100KDa
70KDa
MMP-2
40KDa
Actin
OS-RC-2
Vector
CHIPOE
si-Ctrl
si-CHIP
100KDa
MMP-9
70KDa
MMP-2
40KDa
Actin

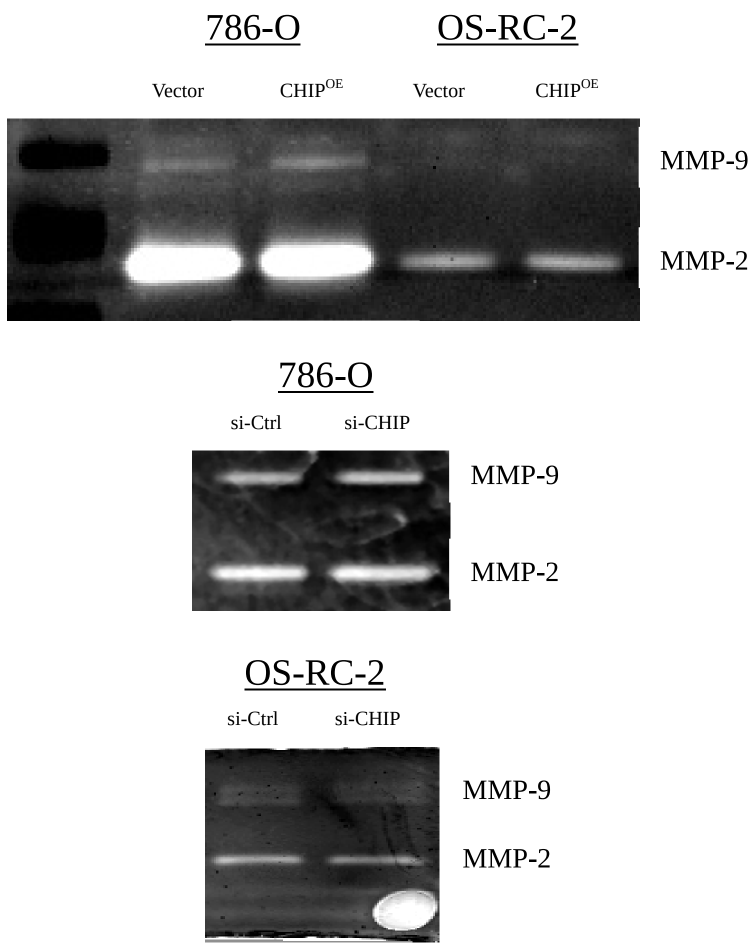
786-O
OS-RC-2
Vector
CHIP^{OE}
Vector
CHIP^{OE}
MMP-9
MMP-2
786-O
si-Ctrl
si-CHIP
MMP-9
MMP-2
OS-RC-2
si-Ctrl
si-CHIP
MMP-9
MMP-2

Figure S5

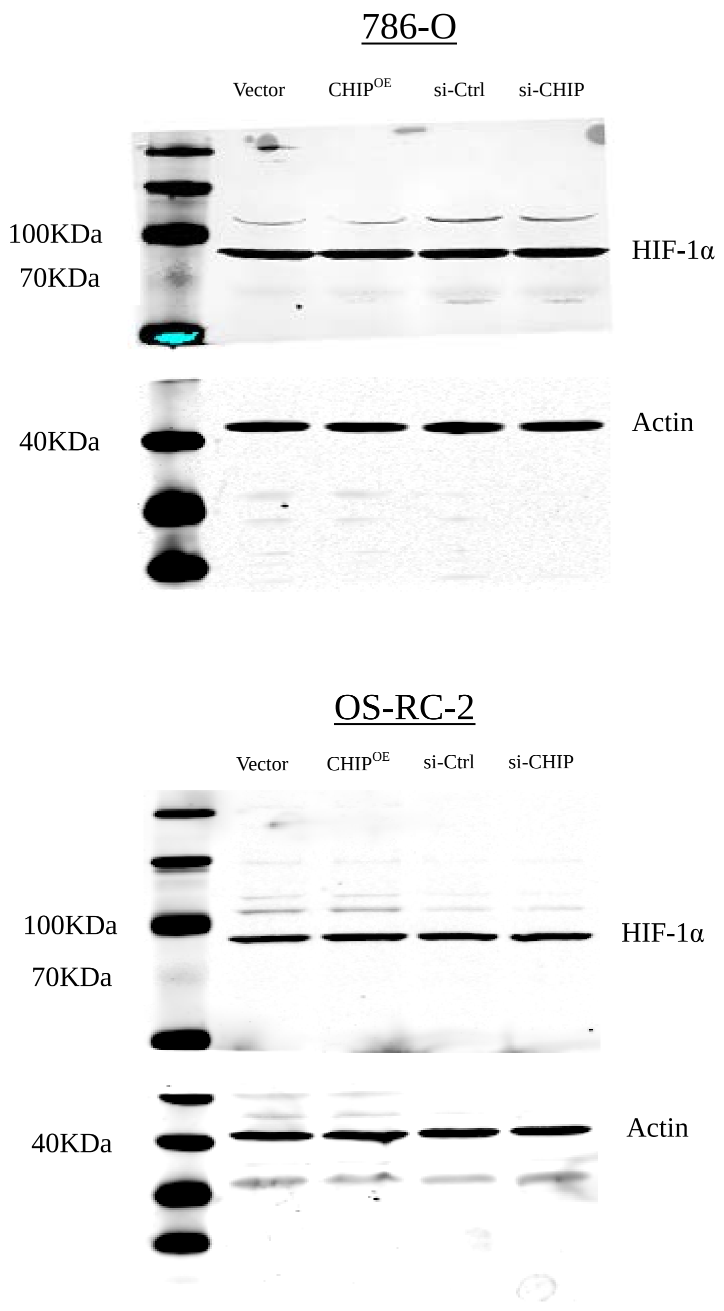
786-O
Vector
CHIPOE
si-Ctrl
si-CHIP
100KDa
70KDa
HIF-1α
40KDa
Actin
OS-RC-2
Vector
CHIPOE
si-Ctrl
si-CHIP
100KDa
70KDa
HIF-1α
40KDa
Actin
